# Feasibility of telemedicine research visits in people with Parkinson’s disease residing in medically underserved areas

**DOI:** 10.1017/cts.2022.459

**Published:** 2022-09-12

**Authors:** Tuhin Virmani, Mitesh Lotia, Aliyah Glover, Lakshmi Pillai, Aaron S. Kemp, Anu Iyer, Phillip Farmer, Shorabuddin Syed, Linda J. Larson-Prior, Fred W. Prior

**Affiliations:** 1 Department of Neurology, University of Arkansas for Medical Sciences, Little Rock, AR, USA; 2 Department of Biomedical Informatics, University of Arkansas for Medical Sciences, Little Rock, AR, USA; 3 Department of Psychiatry, University of Arkansas for Medical Sciences, Little Rock, AR, USA; 4 Little Rock Central High School, Little Rock, AR, USA

**Keywords:** Telemedicine, health equity, rural health, ambulatory monitoring, Parkinson’s disease, medically underserved area

## Abstract

**Introduction:**

Gait, balance, and cognitive impairment make travel cumbersome for People with Parkinson’s disease (PwPD). About 75% of PwPD cared for at the University of Arkansas for Medical Sciences’ Movement Disorders Clinic reside in medically underserved areas (MUAs). Validated remote evaluations could help improve their access to care. Our goal was to explore the feasibility of telemedicine research visits for the evaluation of multi-modal function in PwPD in a rural state.

**Methods:**

In-home telemedicine research visits were performed in PwPD. Motor and non-motor disease features were evaluated and quantified by trained personnel, digital survey instruments for self-assessments, digital voice recordings, and scanned and digitized Archimedes spiral drawings. Participant’s MUA residence was determined after evaluations were completed.

**Results:**

Twenty of the fifty PwPD enrolled resided in MUAs. The groups were well matched for disease duration, modified motor UPDRS, and Montreal Cognitive assessment scores but MUA participants were younger. Ninety-two percent were satisfied with their visit, and 61% were more likely to participate in future telemedicine research. MUA participants traveled longer distances, with higher travel costs, lower income, and education level. While 50% of MUA participants reported self-reliance for in-person visits, 85% reported self-reliance for the telemedicine visit. We rated audio-video quality highly in approximately 60% of visits in both groups. There was good correlation with prior in-person research assessments in a subset of participants.

**Conclusions:**

In-home research visits for PwPD in MUAs are feasible and could help improve access to care and research participation in these traditionally underrepresented populations.

## Introduction

The cost of care for people with Parkinson’s disease (PwPD) is growing with the aging population. A recent study estimated the annual cost of care for PwPD in the US at $51.9 billion, with $25.4 billion attributable to direct medical costs [1]. Additionally, the continued COVID pandemic has highlighted the deficiencies of a clinical model that requires clinical visits to be performed in person [2]. While care from a neurologist improves outcome in PwPD [3], access to specialty care is a significant issue. In Arkansas, a primarily rural state, 3 of the 4 movement disorders trained neurologists practice at a single institution, and approximately 75% of our clinic’s population reside in designated medically underserved areas (MUAs). The driving limitations imposed by motor and cognitive impairment in PwPD [4] limit patients’ independence and make them reliant on spouses, younger family members, or community contacts to shuttle them for clinic visits. The long distances traveled and the time involved to obtain medical care place additional social and psychological burden on patients. Distance to travel is also often reported to us as a limitation to enrollment in research protocols. Objective, secure, and reliable methods of tracking disease progression in the home setting could improve access to care and mitigate some of the costs of care [2,5], even though they may not completely replace in-person care [6].

Although the COVID pandemic overall led to a widely increased utilization of telemedicine for clinical evaluations in movement disorders [7], several concerns have been raised regarding the adoption of more widespread digital technology in general for clinical care [6,8]. People residing in areas with limited cellular or high-speed Internet connectivity or those with low socioeconomic status may have limited access to care [9,10], thereby widening the so-called digital divide. This is an important issue to consider in Arkansas where a significant percentage of the state does not have access to broadband internet access [11]. Additionally in older adults, such as PwPD, ability to use unfamiliar devices could lead to more frustration instead of improved patient outcomes.

Studies have explored the use of telemedicine for clinical health and research visits in PwPD [12–16], but the cohorts have primarily been selected from highly motivated individuals who recently participated in clinical trials [15] or had indicated interest in research participation by signing up in a registry [12–14]. Additionally, only a limited set of assessments were performed in these studies. Our goal in this study was therefore to determine the feasibility of performing a comprehensive set of objective assessments used in routine clinical care and research studies in a population of PwPD residing in a rural state. We also wanted to determine if the ability to perform assessments was different in participants residing in designated MUAs compared to those that did not. Lastly, we wanted to determine if the results of assessments performed remotely were comparable to in-person assessments, and we used two different methods of comparison. Some PwPD develop freezing of gait over the course of their disease progression [17]. Along with other groups, we have shown that PwPD with freezing of gait have differences in disease and gait features outside of the actual episodes of gait freezing [18–31]. We therefore performed a subgroup analysis using the presence or absence of freezing to split groups and compare to differential results previously reported in these sub-phenotypes of PwPD. We also compared the results of in-home and in-person assessments in those participants who had previously participated in in-person research studies in our lab.

## Materials and Methods

### Standard Protocol Approvals, Registrations, and Patient Consents

Participants were recruited from the Movement Disorders Clinic (MDC) at the University of Arkansas for Medical Sciences (UAMS) after the approval of the UAMS institutional review board (IRB#261021). All subjects met UK brain bank criteria for the diagnosis of Parkinson’s disease based on evaluation by a Movement Disorders trained neurologist (TV or ML).

While this project was partly conceived prior to and during the early stages of the COVID pandemic, all participants were enrolled and performed study assessments during the COVID pandemic, between November 2020 and July 2021. Participants were approached during their regularly scheduled clinic visits, and the study was explained in more detail to those who indicated interest. Consent forms were provided to participants for review, after which they were contacted to determine if they would participate. Those agreeing to participate were mailed a research packet approximately one week prior to their scheduled research visit with information regarding setup for the visit, administrative forms (including written informed consent forms), and forms requiring written responses. A return pre-paid envelope was provided for return of these packets. Informed consent was obtained over video from all participants before study assessments were performed, and written signatures were obtained via screen capture and subsequent return of signed forms.

All participants were evaluated at home via the web-based televideo service Doxy.me®, or Doximity® as a backup. The Doxy.me platform allowed multiple users to connect to a single session (i.e., patient, clinician, and research assistant), allowed screen sharing, and allowed patient invitation to the visit via an emailed link (allowing computer use) or a cellular text message link. At the time, Doximity did not have multi-user capability and only allowed participants invitation through cellular text messages and therefore was used as a backup if the Doxy.me platform did not connect with a particular user. Only one participant required the Doximity platform for their visit.

Instruments were created in the Research Electronic Data Capture database (REDCap). To minimize enrollment bias, the designation of medically underserved area (MUA) status of participants, using their home addresses, was performed after study visit completion. Distance to travel was calculated using the participant’s home address and the address for the UAMS MDC.

### Study Assessments

The assessments performed in the study, including mode and reasons for collection, are outlined in Table 1 and discussed in more detail below.

**Table 1.** Remote assessments performed

Assessment	Collection mode	Collected by	Reason for collection
Medications, allergies (5 minutes)^1^	EHR	MD	Treatments for PD
Modified UPDRS (10–15 minutes) ^1^	REDCap	MD	Standard PD scale
N-FOG-Q (1–3 minutes) ^1^	REDCap		Freezing of gait in PD
MoCA [34] (10–15 minutes) ^2^	REDCap	RA	Cognitive decline in PD
Orthostatic vital signs (1 minute) ^1^	EHR/REDCap	P	Autonomic dysfunction in PD
3 m TUG x3 (2–3 minutes)^2^	REDCap	RA/MD	Gait affected in PD
PDQ-39, RBD-Q, ESS [44], (10–12 minutes)^2^	REDCap	P	Sleep and quality of life in PD
Voice sample (5 minutes) ^2^	Secure digital voicemail	P	Voice changes in PD
Handwriting, spiral samples (2–3 minutes) ^2^	Paper	P/RA	Micrographia and spiral asymmetry in PD
Research participation survey (5 minutes) ^2^	REDCap	P	Participant research perspective

Abbreviations: EHR: electronic health record; ESS: Epworth Sleepiness scale; MD: movement disorders neurologist; MoCA: Montreal Cognitive Assessment; N-FOG-Q: New Freezing of gait questionnaire; P: participant; PDQ-39: Parkinson’s disease Quality of Life questionnaire-39; RA: research assistant; RBD-Q: REM sleep disorder questionnaire; TUG: Timed-up-and-go; UPDRS: Unified Parkinson’s Disease Rating Scale.

1 Routine clinical care assessment.

2 Research assessment.

#### Standard of care assessments

A previously validated modified version of the Unified Parkinson’s disease Rating Scale (UPDRS) [32] that excludes the motor assessments of tone (UPDRS item 22) and balance (UPDRS item 30) was utilized. Data were directly entered into REDCap during the video examination (by TV and ML). Medications and allergies were recorded on participant interview or through the UAMS electronic medical record. Orthostatic vitals were obtained by participants who had blood pressure cuffs at home, with explicit written and verbal instructions on correct performance.

#### Freezing of gait determination and quantification

The video for the new freezing of gait questionnaire (N-FOGQ) [33] was shown to all participants via screenshare, and the questionnaire was completed by the neurologists. Participants with a score of 0 on item 1 of the N-FOGQ were assigned to the non-freezers group and those with a score of 1 to the freezers group.

#### Cognitive function

Cognitive function was assessed using the Montreal Cognitive Assessment (MoCA) [34], which has previously been remotely administered [35–37], including in PwPD [38]. For our study, the visuospatial sections of the MoCA were performed as follows. Participants were shown an image of the “trails” task on their screens and asked to verbalize the sequence rather than draw lines on paper. Participants still needed to visualize the sequence and report it correctly. Participants were shown the “cube” image by screenshare and asked to draw the figure on a blank sheet of paper. Instructions were provided for clock drawing as they would be in-person, and participants drew the clock on the provided blank sheet of paper. Immediately after cube and clock drawing, participants held up their drawings to their camera for screen capture and real-time scoring. The written hard copies were also mailed back to us. The animal pictures were screenshared, and participants were asked to name them. Due to visuospatial and executive function impairment being prominent in PwPD, the blind-MoCA, which excludes these important distinguishing features, was not used.

#### Handwriting analysis

Handwriting samples were obtained with a Pilot G2 ball point pen mailed to participants, on a pre-printed sheet that included instructions and spaces to write the sentence “There are earthquakes in California” three times and perform Archimedes spirals with the right and left hand. Participants were asked to angle their camera to allow the examiner to visualize the writing and ensure spirals were drawn without resting arms on the table. A screen capture of the writing samples was obtained immediately to verify correct performance of the tasks. The hard-copy writing sheets were mailed to us and were subsequently scanned, digitized, and used for spiral analysis. Image processing codes were written in Python programming language. OpenCV (Open Source Computer Vision) and NumPy libraries were used to obtain the spiral length, width, and the total distance traveled by the pen tip during spiral drawing. The spiral area was calculated as an ellipse using the formula

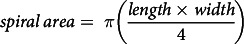




#### Gait measurement

Gait was assessed using the Timed-up-and-go test (TUG). Participants had pre-visit instructions to measure a 10-ft distance, place a chair at one end, and a tape marker at the other end. During the visit, participants were asked to either prop their device or have family members or caregivers hold their device, so that the camera showed the walking area. Participants were instructed when to start and were timed from start until they sat back in the chair. The average of three trials was used for analysis. Participants were allowed to use their assistive devices such as a cane or walker if they routinely utilized these at home.

#### Speech/voice analysis

Voice samples were collected using a secure voicemail that automatically digitized the sample into a *.wav file. Participants were asked to say the sound Ahh for approximately 3 seconds and then read the Rainbow passage [39] aloud. The *.wav files were imported into Audacity®, a free open-source package, to remove background noise (Effect → Noise Reduction) and to split each *.wav file into two separate files for the Ahh sound and the Rainbow passage. Parselmouth [40] (a Python library for acoustical analysis) was used to perform preliminary analysis for basic voice features. The Ahh sound has previously been used in voice analysis in PwPD [41]. The Rainbow passage was additionally used as it has been suggested to be phonetically balanced and allows evaluation of more complex speech patterns. Recorded waveforms were filtered using floor and ceiling values of 75 decibels (dB) and 300 dB respectively for males, and 100 dB and 600 dB for females. Several features were extracted from the waveforms and analyzed as they have been previously shown to be affected in PwPD [42]. The mean fundamental frequency (f0) describes the pitch of sound, while the standard deviation in f0 describes the variability in pitch. Local “jitter” is defined as the frequency variation in f0 from cycle to cycle and provides a measure of the extent of variation in voice range and vocal tremor [42]. Shimmer describes the amplitude variation of the sound wave in each vocal cycle, thereby providing insight into the hypophonia and variations in voice amplitude that can occur in PwPD [42]. The mean Harmonics to Noise Ratio (HNR), defined as the ratio of noise to tonal speech, is due to incomplete vocal cord closure [42].

#### Other scales

REDCap survey instruments were developed for participants to complete the self-filled Parkinson’s disease quality of life scale-39 (PDQ-39) [43] and two sleep scales, the Epworth sleepiness scale (ESS) [44] and the REM sleep behavior disorder questionnaire (RBD-Q) [45].

#### Telemedicine surveys

At the completion of their visit, participants were asked to complete a survey to gauge their satisfaction with the visit and their perception of audio-video quality. Optionally, they were asked to provide their annual income and estimated costs to attend in-person visits. The research team also completed a survey to document audio-video quality, perceived issues, and time taken to perform assessments over telemedicine compared to in-person research assessments. The total time for each visit was also recorded.

### In-person Comparison Group

A group of 26 participants enrolled in this telemedicine study had previously undergone in-person research assessments, pre-COVID pandemic, as part of an IRB-approved protocol (UAMS IRB#203231). This protocol allowed use of their data for analysis in future studies. The results of the previously administered in-person UPDRS, MoCA, RBD-Q, PDQ-39 scores, and TUG times in these individuals were compared to the equivalent remotely administered assessments. For the in-person UPDRS, only the components that could be administered remotely were used.

### Statistical Analysis

Statistical analysis was performed using SPSS version 25 (IBM). Normality was tested using the Shapiro-Wilk test for each assessment. Normally distributed variables included age at enrollment, N-FOG-Q, spiral width and height, spiral pen distance traveled, AAH sound mean f0 and HNR, and RP local shimmer and HND (Table 2). To assess group differences between MUA and non-MUA groups and freezers and non-freezers, one-way analysis of variance (ANOVA) was used for normally distributed variables, and the non-parametric Mann-Whitney U-test was used for non-normally distributed variables. A Benjamini-Hochberg correction was applied to the groups of voice and handwriting analysis features independently. Kendall’s tau-b correlation coefficients, with a Benjamini-Hochberg correction, were used to determine the associations between disease measures (age, disease duration, motor, and total UPDRS scores, H&Y scores, MoCA, average TUG time, and PDQ-39 scores) and voice analysis features. Intraclass correlation coefficients (ICC) were calculated, and Bland-Altman plots were used to determine the association between the in-home assessments with in-person assessments in a subset of participants who participated in prior IRB-approved research in our lab (n = 26). ICC allowed comparison of our results to historical literature that validated these assessments, while the Bland-Altman plots allowed easier determination of whether repeated results significantly differed from one another, along with a clearer picture of the variability of the data set. For survey results, Pearson’s chi-square was used to determine statistical difference between the MUA and non-MUA groups for questions with responses classified as nominal variables (Table 3 Items 3, 4, 6, 7 & Table 4 Items 5, 6, 10, 11), while the Mann-Whitney U-test was used for responses classified as ordinal variables (remaining Table 3 and 4 Items). A generalized linear repeated measures analysis was used with the interaction effects between location (in-person vs in-home) and group (MUA vs non-MUA) being the variable of interest to determine if participant’s self-reliance was different.

**Table 2.** Participant demographics and assessment results

	All PwPD (n = 50)	Reside in MUA (n = 20)	Not in MUA (n = 30)
Sex (Female/male)	30/20	11/9	19/11
Education (years)	16.3 ± 2.4	15.2 ± 2.2	17.0 ± 2.2
Race (Caucasian %)	100%	100%	100%
Age at enrollment (years)	65.8 ± 9.2	61.9 ± 9.3	68.4 ± 8.4*
Disease duration (years)	9.2 ± 5.7	8.7 ± 4.8	9.6 ± 6.3
Hoehn & Yahr stage	2.0 ± 0.5	2.0 ± 0.6	2.0 ± 0.3
Modified motor UPDRS	12.5 ± 6.4	11.2 ± 5.6	13.4 ± 6.9
Modified total UPDRS	24.0 ± 10.7	22.7 ± 11.6	24.9 ± 10.1
MoCA score	26.1 ± 2.9	26.4 ± 2.2	26.0 ± 3.3
N-FOG-Q score	14.2 ± 6.5 (n = 17)	15.1 ± 6.8 (n = 9)	13.1 ± 6.4 (n = 8)
PDQ-39	27.5 ± 21.3	33.7 ± 26.2	23.4 ± 16.7
RBD-Q	5.0 ± 3.1	5.2 ± 3.2	4.8 ± 3.2
Epworth Sleepiness Scale	7.7 ± 4.9	9.8 ± 6.1	6.2 ± 3.5^#^
Daily levodopa dose (mg)	676 ± 318	650 ± 270	695 ± 350
On agonist/MAO-I	28%/40%	40%/40%	20%/40%
Mean 10 ft TUG time (s)	11.9 ± 3.1	11.9 ± 2.9	12.0 ± 3.4
Distance from UAMS; 25/50/75 percentile (miles)	8/40/101	37/65/114	8/12/55^#^
*Vitals*	n = 40	n = 14	n = 26
Mean seated BP, HR	123/77, 76	127/80, 77	121/76, 75
Mean standing BP, HR	123/81, 81	126/83, 80	121/80, 81
% orthostatic on BP	5%	0%	7%
% orthostatic on HR	8%	0%	12%
*Spiral analysis (scanned hard-copy)*			
Disease MA spiral height (cm)	4.8 ± 1.1	4.8 ± 1.1	4.8 ± 1.1
Disease MA spiral width (cm)	5.3 ± 1.5	5.3 ± 1.6	5.3 ± 1.5
Disease MA spiral area (cm^2^)	20.6 ± 9.8	20.6 ± 9.5	20.7 ± 10.2
Disease MA spiral pen travel dist. (cm)	37.8 ± 12.0	36.0 ± 10.8	39.1 ± 12.7
*Voice analysis (digital voicemail)*	n = 49	n = 19	n = 30
*Ahh* sound duration (s)	3.8 ± 1.8	3.3 ± 1.6	4.0 ± 1.9
*Ahh* sound f0 mean (Hz)	179.3 ± 44.8	178.9 ± 51.0	179.7 ± 41.4
*Ahh* sound f0 standard deviation (Hz)	9.0 ± 13.4	5.6 ± 6.3	11.2 ± 16.1
*Ahh* sound local Jitter (%)	0.7 ± 0.5	0.6 ± 0.3	0.7 ± 0.5
*Ahh* sound local Shimmer (%)	6.7 ± 2.5	6.1 ± 2.2	7.1 ± 2.6
*Ahh* sound HNR (dB)	17.7 ± 3.5	17.9 ± 3.4	17.5 ± 3.6
	n = 48	n = 19	n = 29
*Rainbow passage* (RP) duration (s)	135.4 ± 41.7	133.8 ± 45.6	136.5 ± 39.7
*RP* f0 mean (Hz)	163.4 ± 40.4	164.2 ± 41.7	162.8 ± 40.3
*RP* f0 standard deviation (Hz)	37.3 ± 19.0	37.0 ± 19.2	37.5 ± 19.2
*RP* local Jitter (%)	2.2 ± 0.5	2.1 ± 0.5	2.2 ± 0.6
*RP* local Shimmer (%)	11.6 ± 1.6	11.4 ± 1.2	11.7 ± 1.9
*RP* HNR (dB)	12.4 ± 2.3	12.2 ± 2.0	12.6 ± 2.5

Abbreviations: BP: blood pressure; HNR: harmonics to noise ratio; HR: heart rate; MA: more affected; MoCA: Montreal Cognitive Assessment; MAO-I: monoamine oxidase inhibitors; MUA: medically underserved area; N-FOG-Q: New Freezing of gait questionnaire; PDQ-39: Parkinson’s disease Quality of Life questionnaire-39; PwPD: People with Parkinson’s disease; RBD-Q: REM sleep disorder questionnaire; TUG: Timed-up-and-go; UAMS: University of Arkansas for Medical Sciences; UPDRS: Unified Parkinson’s Disease Rating Scale.

* p < 0.05 between MUA and non-MUA by ANOVA.

# p < 0.05 between MUA and non-MUA by Mann-Whitney test.

**Table 3.** Participant survey

	All PwPD (n = 50)	Reside in MUA (n = 20)	Not in MUA (n = 30)
1. Scheduling appointment was easy:			
Strongly agree	86%	90%	83%
Somewhat agree	14%	10%	17%
Neither agree nor disagree	0%	0%	0%
Somewhat disagree	0%	0%	0%
Strongly disagree	0%	0%	0%
2. I was happy with my telemedicine visit:			
Strongly agree	92%	85%	97%
Somewhat agree	6%	15%	0%
Neither agree nor disagree	0%	0%	0%
Somewhat disagree	2%	0%	3%
Strongly disagree	0%	0%	0%
3. What did you like about the telemedicine visit:			
No travel arrangements	70%	75%	67%
Ability to be in comfort of your home	84%	80%	87%
Ability to participate in research	82%	75%	87%
4. What did you dislike about the telemedicine visit:			
Poor video connection	6%	10%	3%
Unable to hear provider	22%	20%	23%
Poor internet connection	6%	10%	3%
Prefer in-person visit	28%	35%	23%
5. More likely to participate in telemedicine research in the future:			
Strongly agree	29%	35%	24%
Somewhat agree	33%	30%	34%
Neutral	33%	30%	34%
Somewhat disagree	2%	5%	0%
Strongly disagree	4%	0%	7%
6. Whom do you rely on for in-person visits? (check all that apply)			
Self	64%	50%^^^	73%
Spouse	52%	65%	43%
Children	6%	10%	3%
Others	2%	5%	0%
7. Whom did you rely on for telemedicine visit? (check all that apply)			
Self	80%	85%^^^	77%
Spouse	34%	40%	30%
Children	2%	0%	3%
Others	6%	10%	3%
8. Overall visit rating:			
Extremely bad	0%	0%	0%
Bad	0%	0%	0%
Neutral	0%	0%	0%
Good	27%	25%	28%
Excellent	73%	75%	72%
9. Annual Income:			
<$25,000	5%	11%^#^	0%
$25–50,000	16%	21%	13%
$50–75,000	16%	26%	8%
$75–100,000	14%	11%	17%
>$100,000	49%	32%	63%
10. Costs to attend in-person visit:			
<$35	56%	26%^#^	79%
$36–75	19%	26%	13%
$76–150	12%	21%	4%
$151–300	9%	21%	0%
>$300	5%	5%	4%

Abbreviations: MUA: medically underserved area; PwPD: People with Parkinson’s disease.

^ p < 0.05 repeated measures analysis for self-reliance between in-person and telemedicine visit.

# p < 0.05 Mann-Whitney U-test.

**Table 4.** Research survey

	All PwPD (n = 50)	Reside in MUA (n = 20)	Not in MUA (n = 30)
Total visit time (hours)	1.4 ± 0.4	1.4 ± 0.3	1.4 ± 0.4
Extra time required to setup clinic visit			
<5 minutes	80%	75%	83%
5–15 minutes	16%	20%	13%
16–30 minutes	4%	5%	3%
31–45 minutes	0%	0%	0%
>45 minutes	0%	0%	0%
Extra time required for clinic assessments			
<5 minutes	94%	95%	93%
5–15 minutes	6%	5%	7%
16–30 minutes	0%	0%	0%
31–45 minutes	0%	0%	0%
>45 minutes	0%	0%	0%
Issues with a particular clinic assessment			
No problems	94%	90%	97%
One or more assessments	4%	10%	0%
Entire visit	2%	0%	3%
Specific clinical assessments with issues			
Vitals	0%	0%	0%
Medications	0%	0%	0%
N-FOG-Q	0%	0%	0%
UPDRS	4%	10%	0%
TUG	2%	0%	5%
Audio-video quality clinical assessment			
Great	60%	50%	67%
Video a little slow	30%	35%	27%
Video quality mixed	6%	10%	3%
Video details barely visible	4%	10%	0%
Video dropping connection	0%	0%	0%
No audio	0%	0%	0%
Audio-video mismatch	4%	0%	7%
Audio only, no video	0%	0%	0%
Audio by telephone	0%	0%	0%
Barely audible	0%	0%	0%
Extra time required to setup research visit			
<5 minutes	86%	85%	86%
5–15 minutes	14%	15%	14%
16–30 minutes	0%	0%	0%
31–45 minutes	0%	0%	0%
>45 minutes	0%	0%	0%
Extra time required to for research assessments			
<5 minutes	58%	65%	53%
5–15 minutes	18%	15%	20%
16–30 minutes	10%	10%	10%
31–45 minutes	2%	5%	0%
>45 minutes	12%	5%	17%
Issues with a particular research assessment			
No problems	78%	70%	83%
One or more assessments	20%	25%	17%
Entire visit	2%	5%	0%
Specific research assessments with issues			
MoCA-any component	18%	25%	13%
MoCA-visuospatial	10%	10%	10%
MoCA-other	8%	15%	3%
Handwriting	0%	0%	0%
Speech	2%	0%	3%
PDQ-39	0%	0%	0%
RBD-ESS	2%	5%	0%
Telemedicine survey	0%	0%	0%
Audio-video quality research assessments			
Great	62%	60%	63%
Video a little slow	20%	15%	23%
Video quality mixed	8%	10%	7%
Video details barely visible	4%	10%	0%
Video dropping connection	2%	0%	3%
No audio	0%	0%	0%
Audio-video mismatch	6%	0%	10%
Audio only, no video	0%	0%	0%
Audio by telephone	6%	5%	7%
Barely audible	2%	5%	0%

Abbreviations: ESS, Epworth sleepiness scale; MoCA: Montreal Cognitive Assessment; MUA: medically underserved area; N-FOG-Q: New Freezing of gait questionnaire; PDQ-39: Parkinson’s disease Quality of Life questionnaire-39; PwPD: People with Parkinson’s disease; RBD: REM sleep behavior disorder; TUG: Timed-up-and-go; UPDRS: Unified Parkinson’s Disease Rating Scale.

### Data Sharing

The data from this manuscript will be available in a public repository on acceptance of the manuscript. To support optimal data integration for analysis, we combined all study data into a single collection using the Arkansas Research Image Enterprise System (ARIES) [46,47]. ARIES supports integration of multimedia data, including sound files, and extracts from both the REDCap database and the UAMS Arkansas Research Clinical Data Repository (AR-CDR) [48,49]. All ARIES data are de-identified using an integrated utility [49] to facilitate use by the study team and potential reuse by other researchers. Data for a given patient can be added using a secure record linkage mechanism connecting ARIES data instances with data pulled from the AR-CDR.

## Results

**Fig. 1.**
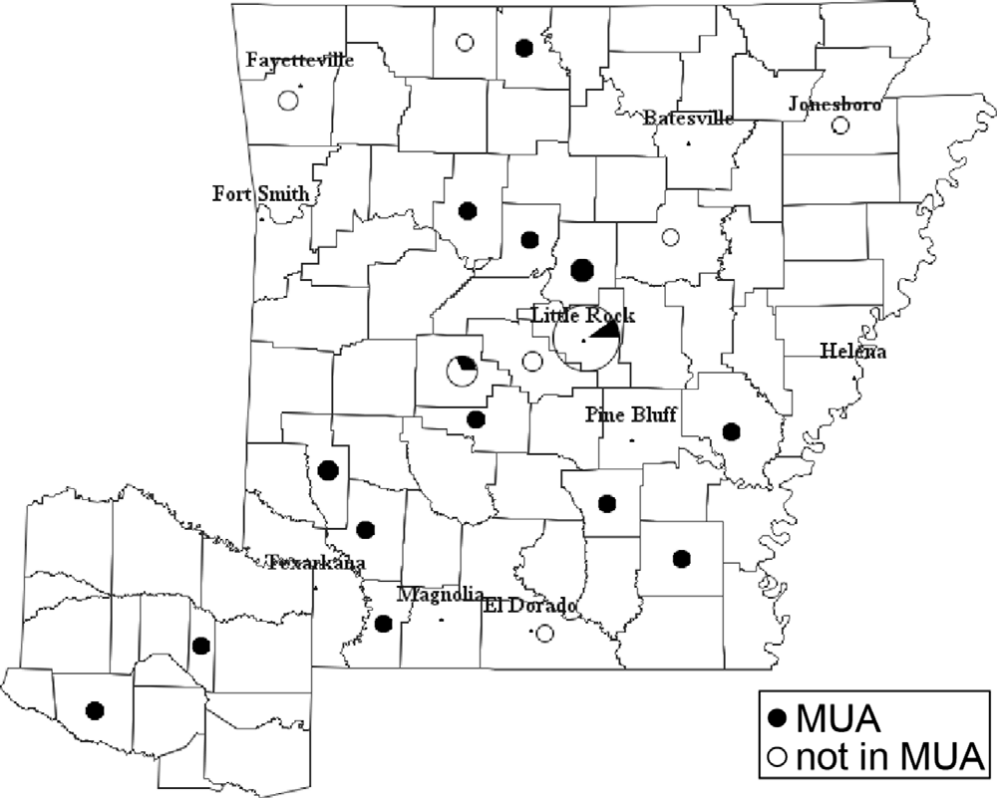
Participant distribution: distribution of 50 participants and their residence in medically underserved areas (MUA, filled area of circle) or not (non-MUA, un-filled area of circle).

The demographics and assessment results of all participants are shown in Table 2. Based on their home address, 20 participants resided in MUAs (Fig. 1). Participants in the MUA and non-MUA groups had similar disease duration, modified UPDRS motor and total scores, N-FOG-Q scores, and gender distribution. The MUA participants were younger at enrollment. On the TUG, walking speed was similar in both groups, based on similar times to walk a 10-ft distance. Cognition using the MoCA was similar between groups. Sleep quality was variable, with similar rates of REM sleep behavior disorder based on the RBD-Q scores, but greater daytime sleepiness in the MUA group based on the ESS scores. This could partially be due to higher dopamine agonist usage in the MUA group as those on agonists had higher RBD-Q scores (4.3 ± 2.8 not on agonist, 6.7 ± 2.8 on agonist, p = 0.015 ANOVA) and trended toward higher ESS scores (7.0 ± 4.3 not on agonist, 9.7 ± 6.1 on agonist, p = 0.080 ANOVA). While quality of life scores (PDQ-39) was worse in those residing in MUAs, this was not statistically significant. MUA participants resided significantly further away from UAMS than non-MUA participants (Table 2).

The hard-copy samples of the Archimedes spiral drawings collected on paper that were mailed to us by participants were digitized and analyzed using in-house Python scripts. Consistent with similar motor UPDRS scores and Timed-Up-and-Go (TUG) times, there were no differences in spiral measures between the MUA and non-MUA groups (Table 2).

Voice samples were successfully collected using digital voicemail, with no notable differences in the quality of samples obtained from those residing in MUAs. Preliminary voice acoustical analysis using Parselmouth [40] showed no differences in standard voice features between MUA and non-MUA participants. After application of a Benjamini-Hochberg correction for multiple comparisons, RP duration was inversely correlated with MoCA scores (−0.402, p = 0.0001).

### Comparison to Prior In-person Research Results

**Fig. 2.**
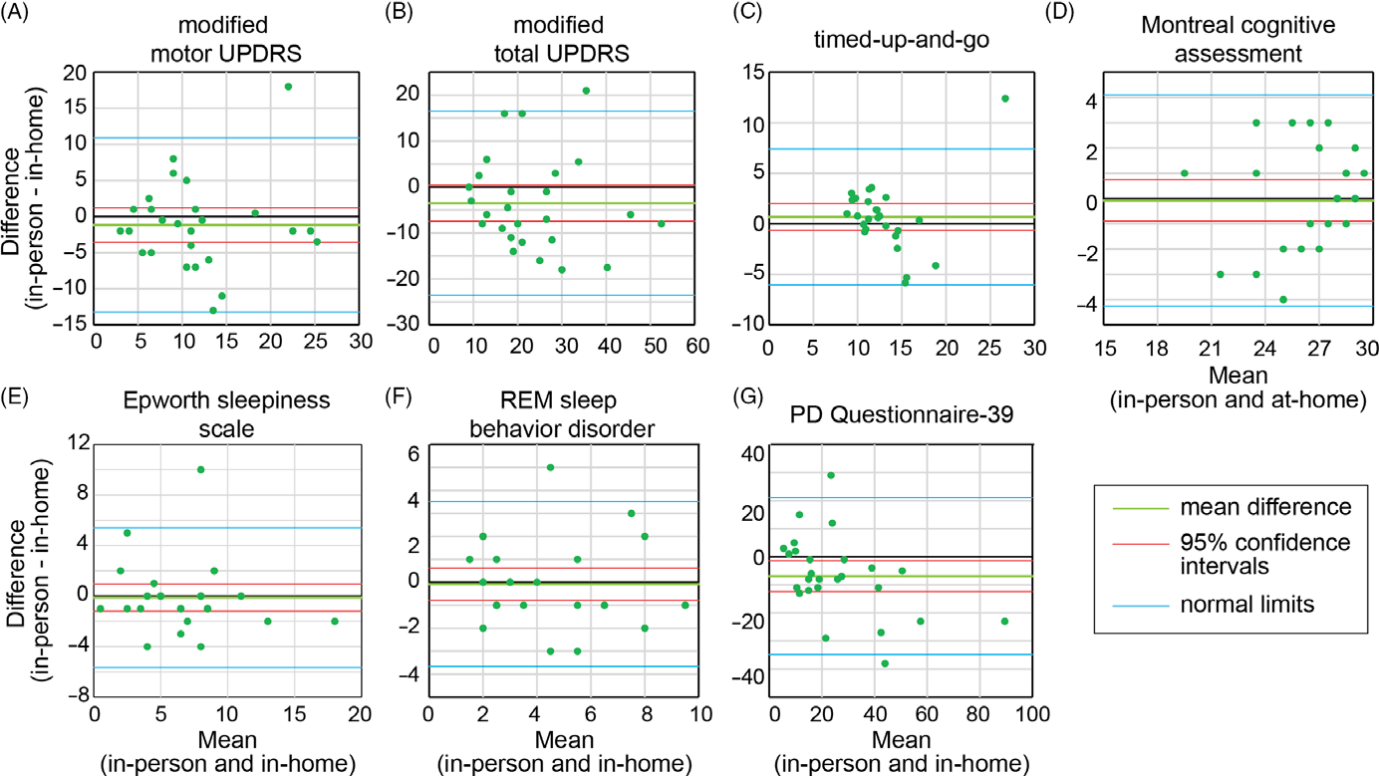
Agreement between in-home and in-person assessments. Bland-Altman plots comparing the results of the two different modes of assessments for the (A) modified motor Unified Parkinson’s Disease Rating Scale (UPDRS), (B) modified total UPDRS, (C) timed-up-and-go, (D) Montreal Cognitive Assessment, (E) Epworth Sleepiness scale, (F) REM Sleep Behavior Disorder questionnaire, and (G) the Parkinson’s Disease (PD) Questionnaire-39. Plots with the zero line (black) between the 95% confidence intervals (red) show agreement between the results.

Twenty-six participants had previously performed in-person research assessments, approximately 1.4 ± 0.4 years prior to their in-home visits, prior to the COVID pandemic. To determine validity of our in-home measurements, we compared the results in these individuals between the two modes of participation. Bland-Altman plots (Fig. 2) showed agreement between these measures, except in the case of the PDQ-39, where the 0 line lies outside of the 95% confidence intervals. There was significant correlation in all measures (Supplementary Table 1) with ICC ranging from 0.757 (motor UPDRS) to 0.870 (RBD-Q).

### Subgroup Analysis of Freezers and Non-freezers

As an additional test of validity of our in-home assessments, we split our study population into freezers (n = 17) and non-freezers (n = 33) using item 1 of the N-FOG-Q, and results are provided in Table 5. Consistent with previous findings [28], freezers had longer disease duration (7.3 ± 4.1; 12.9 ± 6.7), higher total UPDRS scores (21.3 ± 9.1; 29.1 ± 11.9), and worse quality of life scores on the PDQ-39 (19.7 ± 12.2; 42.7 ± 27.0).

**Table 5.** Freezers vs non-freezers demographics and assessment results

	Non-freezers (n = 33)	Freezers (n = 17)
Sex (female/male)	11/22	9/8
Age at enrollment (years)	66.7 ± 10.2	64.1 ± 7.1
Disease duration (years)	7.3 ± 4.1	12.9 ± 6.7^#^
Hoehn & Yahr stage	1.9 ± 0.4	2.2 ± 0.5^#^
Modified motor UPDRS	12.0 ± 6.4	13.5 ± 6.6
Modified total UPDRS	21.3 ± 9.1	29.1 ± 11.9^#^
MoCA score	25.9 ± 3.0	26.5 ± 2.6
N-FOG-Q score	–	14.2 ± 6.5
PDQ-39	19.7 ± 12.2	42.7 ± 27.0^#^
RBD-Q	4.6 ± 3.0	5.7 ± 3.3
Epworth Sleepiness Scale	7.5 ± 4.3	8.1 ± 6.2
Daily levodopa dose (mg)	613 ± 338	794 ± 241^#^
On agonist/MAO-I	27%/42%	29%/35%
Mean 10 ft TUG time (s)	11.5 ± 2.6	12.8 ± 3.8
*Vitals*	n = 26	n = 14
Mean seated BP, HR	123/77, 76	123/78, 76
Mean standing BP, HR	122/80, 81	124/84, 81
% orthostatic on BP	0%	14%
% orthostatic on HR	8%	8%
*Spiral analysis*		
Disease MA spiral height (cm)	4.9 ± 1.2	4.5 ± 0.9
Disease MA spiral width (cm)	5.5 ± 1.7	4.9 ± 1.2
Disease MA spiral area (cm^2^)	22.3 ± 10.7	18.3 ± 7.1
Disease MA spiral pen travel dist. (cm)	39.1 ± 11.7	35.3 ± 12.4
*Voice analysis*	n = 33	n = 16
*Ahh* sound duration (s)	3.9 ± 2.1	3.5 ± 1.0
*Ahh* sound f0 mean (Hz)	172.9 ± 43.6	192.7 ± 45.9
*Ahh* sound f0 standard deviation (Hz)	8.0 ± 13.0	11.1 ± 14.4
*Ahh* sound local Jitter (%)	0.7 ± 0.5	0.7 ± 0.3
*Ahh* sound local Shimmer (%)	6.7 ± 2.8	6.7 ± 1.8
*Ahh* sound HNR	17.3 ± 3.9	18.4 ± 2.4
	n = 32	n = 16
*Rainbow passage* (RP) duration (s)	128.0 ± 33.3	150.3 ± 53.0
*RP* f0 mean (Hz)	155.4 ± 37.8	179.4 ± 42.0
*RP* f0 standard deviation (Hz)	33.5 ± 15.6	44.8 ± 23.2
*RP* local Jitter (%)	2.1 ± 0.4	2.5 ± 0.6^#^^,^^^^
*RP* local Shimmer (%)	11.6 ± 1.8	11.5 ± 1.4
*RP* HNR	12.2 ± 2.6	12.8 ± 1.7

Abbreviations: BP: blood pressure; HNR: harmonics to noise ratio; HR: heart rate; MAO-I: monoamine oxidase inhibitors; MoCA: Montreal Cognitive Assessment; N-FOG-Q: New Freezing of gait questionnaire; PDQ-39: Parkinson’s disease Quality of Life questionnaire-39; RBD-Q: REM sleep disorder questionnaire; TUG: Timed-up-and-go; UPDRS: Unified Parkinson’s Disease Rating Scale.

# p < 0.05 between freezers and non-freezers by Mann-Whitney U-test.

^ Not significant after Benjamini-Hochberg correction.

### Participant Satisfaction Survey

In a post-study survey (Table 3), 82% of participants reported ability to participate in research as a positive feature of their in-home visit, although when asked what they disliked, 28% responded that they preferred in-person visits. Only 6% disagreed on a question asking if they were more likely to participate in telemedicine research in the future. A greater percentage of participants reported relying on themselves for in-home vs in-person visits (80% vs 64% respectively), and this difference was greater in the MUA group (85% vs 50% respectively). While household income was lower in MUA participants compared to non-MUA (32% vs 13%, respectively, self-reported <$50,000/year), costs to attend traditional in-person visits were higher in the MUA participants (47% vs 8% respectively reporting >$75 to attend). Level of education was lower in the MUA group (Table 2).

### Visit Quality

To determine technical difficulties from the research administration standpoint, the research team completed post-visit surveys (Table 5). The components of the visit considered to be routine clinical care or research assessments (see Table 1, superscript 1 – routine clinical care, superscript 2 – research assessments) were graded separately. Only 14% of participants required >5 minutes on setup for administration of research assessments. While 42% required >5 minutes additional time to help understand, locate, or complete the research assessments compared to our prior experience administering these assessments in-person (Table 4), this was not significantly different between the MUA and non-MUA participants. The total visit time was also similar in both groups. Audio-video quality was rated great for 60% of visits, and there was no significant difference in the audio-video quality between the MUA and non-MUA groups (Table 4).

## Discussion

Due to the COVID-19 pandemic, clinical care was forced to rapidly adapt to the need for restricted travel and in-person interaction to decrease spread of COVID-19. This led to an increased interest in remote assessments of neurologic disorders [50]. In this pilot study, we assessed the viability of enrolling PwPD residing in a predominantly rural state in the United States, in a home-based telemedicine study. We developed digital data collection instruments for validated tools for the assessment of motor and non-motor features of PD and deployed them in this study. We were able to show that people were willing and able to participate in telemedicine-based research, with 40% of our participants residing in MUAs that historically have limited participation in research and telemedicine [51]. We were able to show that age, disease duration, and disease severity-matched participants could be enrolled from MUAs. We show that the quality of audio-video connectivity, even in rural areas, was adequate to implement the routine clinical and research assessments performed on PwPD. We also showed using both subgroup analysis of freezers and non-freezers, and historical in-person research evaluations previously performed on a subgroup of participants, that remote assessment results were valid and reproduced previous findings. We were also able to add new assessments that we had not previously performed such as voice analysis and reproduce the results from other groups.

A few studies have used remote visits to determine feasibility of research participation. Dorsey et al. [14] enrolled participants from the Fox Trial Find registry and performed MoCA and modified MDS-UPDRS measures. Like in our study, overall patient and neurologist satisfaction was high. While the number of participants was three times those in our study, these participants were highly motivated for research participation, having enrolled in a registry indicating interest and therefore were a different population to our rural population, where 40% resided in MUAs, and 50% had not previously participated in research. In a more recent study, Tarolli et al. [15] showed that remote evaluations of PwPD after participation in a clinical trial were also feasible and results showed good correlation with in-person visits. The UPDRS has also been successfully implemented previously in small clinical studies, either via telemedicine alone [12], compared to in-person assessments [16], and in repeated monitoring in the setting of a continuous care facility [52].

A subset of participants in our study had previously performed IRB-approved in-person research studies on average 1.5 years prior to the telemedicine visit. The ICCs between in-home and prior in-person research visits (0.757 UPDRS III and 0.825 MoCA) were better than those previously reported by Tarolli et al. [15] (0.51 UPDRS III and 0.62 MoCA) and Cubo et al. [16] (0.63 UPDRS III) performed closer together. In the initial validation studies for the assessments, the correlation coefficients ranged from 0.68 for the social subscale of the PDQ-39 [43] to 0.92 for the UPDRS [53] and MoCA [34]. It is likely that disease progression and COVID isolation-related superimposed functional limitations could account for some of the variability between our two measurements, especially in quality of life. It might be expected that the 1.5-year interval between assessments in our cohort should decrease correlation (ICC) and mean difference measures (Bland-Altman plots) due to different disease progression rates between participants. This could lead to faster progressing participants having greater differences in these values between the assessments, with slower progressing individuals having lesser differences. This would result in increased scatter in correlation between values and lower correlation coefficients. In our studies, both motor and cognitive assessments were administered to participants while they were in the levodopa medicated state, which could mask significant disease progression. In support of this, in a prior study of disease progression in PwPD with and without freezing, the group without freezing, similar to the majority of participants in the current study, had an average decline of 1 point/year on the motor UPDRS and 0.4 points/year on the MoCA, when longitudinally assessed in the levodopa medicated state [54]. We cannot exclude the possibility that participants with slower disease progression enrolled in this study, although this should not affect the results as the MUA and non-MUA groups were well matched for disease duration, Hoehn and Yahr scores, MoCA scores and motor UPDRS scores with an average of 9 years disease duration and Hoehn and Yahr scores of 2. Repeating this study once the pandemic restrictions allow performance of short interval serial in-person and home-based assessments would likely provide stronger correlations.

Studies of clinical virtual visits have also been performed on a highly motivated population of PwPD who visited a website and submitted interest in participating [12,13]. While there was no improvement in quality of life with in-home over in-person visits [12,13], satisfaction was high among both neurologists and patients in both studies. Other studies using surveys alone also show high satisfaction with clinical virtual visits compared to office visits [55,56]. In our study, in addition to 75% participant satisfaction, importantly, participants felt that they were more self-reliant with the in-home visits compared to historical in-person visits.

The MoCA has been previously used for remote administration in diverse populations [36], including a small study in PwPD [35]. The ability to perform reliable cognitive testing remotely however is still of interest. The results could be “improved” by utilizing people outside of the camera field for help or by taking notes to help improve scores on memory items. To ensure participants were performing their own assessments, we had them angle their camera toward the writing table during writing assessments (cube and clock drawing) and requested they not write down the items to be recalled. Their microphones were on to hear if caregivers provided assistance. Among our instruments, the MoCA provided the greatest difficulty in administration, albeit in only 18% of participants, primarily in the visuospatial domain assessments. In the 26 participants with prior MoCA scores, the correlation with prior administration was high.

There are concerns about remote visits widening the digital divide [57] and our study aimed to provide evidence that remote assessments can be reliable, even in an elderly population living in MUAs in a rural state, thereby improving healthcare equity. Research participation also helps empower people by allowing them to participate in the search for better outcomes for their disease. It is important to note that we did not target recruitment efforts toward enrollment of MUA participants, yet 40% of the participants were from such areas. This remains lower than expected based on the demographics of our clinic population where 75% reside in MUAs. The quality of videoconferencing is one of the concerns commonly expressed about remote visits, and there have been limited small studies evaluating this [58]. We rated the quality of audio-video similarly in both MUA and non-MUA populations, while blinded to participants residence status. In PwPD, given that onset is commonly in later life, elderly patients may be less comfortable accessing new technology needed to perform telemedicine visits, increasing reliance on others to help with medical care. Contrary to this, participants in our study felt more self-reliant with the in-home visits and the majority felt they were more likely to participate in future telemedicine research, independent of their residence location.

About a third of participants reported that they preferred in-person visits (to in-home visits). In a post hoc analysis (Supplementary Table 2), we found no significant differences in age, disease duration, motor UPDRS scores, or TUG times based on this item response, that might suggest less severely affected participants were more willing to travel to visit in-person. Distance traveled for in-person visits was also similar in those preferring in-person visits to those that did not indicate this. However, the participants that suggested preference for in-person visits did appear to have a higher annual income with 64% with incomes >$100,000 and 0% <$50,000, compared to 44% and 28%, respectively, in the group that did not suggest a preference for in-person visits. Further exploration of this income inequality in the MUA population and choice of visit type is warranted, as it suggests that lower income, underserved populations are willing to utilize telemedicine. Those who preferred in-person visits were possibly less reliant on others for their in-person visits and were not as likely to participate in future telemedicine research. It must be noted however that this preference for in-person visits is based on a questionnaire after a research visit that lasted on average 1.4 hours. It is possible that this would be different if only a 30-minute clinical visit was performed. It is important to keep in mind that these data are self-report and must be interpreted with caution.

The medically underserved population in our study had lower self-reported annual income, lower education levels, and traveled longer distances to attend in-person visits. They also reported higher costs to attend in-person visits, which could be secondary to costs associated with travel, meals that were required to be purchased, and potential need for hotel accommodations to break up the journey. Incorporation of telemedicine into clinical and research practice can help reduce costs associated with healthcare. Additionally, the use of telemedicine in this underserved population could greatly improve the ease of access to clinical care, and network-based models focused on patient-centered care have been proposed [5].

One of the limitations of our study was that due to costs, we were unable to incorporate remote sensors for objective evaluation of limb bradykinesia and gait [59]. Future addition of properly validated, inexpensive, and reliable sensors [60,61] for objective remote monitoring of motor function in rural and underserved areas could further extend our results. Performing Lee Silverman Voice Treatment [62], exercise and physical therapy [63] and cognitive behavioral therapy [64] via telemedicine have also been gaining momentum, but need to be tested in underserved populations. Due to the COVID pandemic, our in-person comparisons were limited to only half the participants and were performed 1.5 years prior to the telemedicine assessments. Disease progression could impact our results; however, as discussed in greater detail above, there was still significant agreement between the two assessments performed. These results therefore still provide evidence for the feasibility of conducting such assessments using telemedicine in a manner that tracks disease. Additionally, due to the multiple measured features and possible false discovery rate, caution should be taken to not overinterpret any statistical group differences.

In summary, we show that in-home telemedicine visits can be conducted in PwPD residing in MUAs, that assessments performed show concordance with those performed in-person, and that participants were not only satisfied with the visits and felt more self-reliant with such visits, but that they would be willing to participate in telemedicine-based research in the future. These results provide support for continued incorporation of remote assessments into research studies and clinical care in conjunction with current in-person care models in the future.

## Supplementary material

For supplementary material accompanying this paper visit https://doi.org/10.1017/cts.2022.459. click here to view supplementary material
