# Supplementary material for: Feasibility of telemedicine research visits in people with Parkinson’s disease residing in medically underserved areas
Source: J Clin Transl Sci. 2022 Sep 12;6(1):e133. doi: 10.1017/cts.2022.459 (PMC9794963; doi:10.1017/cts.2022.459)
Supplement: Supplementary file 1 [file S2059866122004599sup001.docx]

| Supplementary Table 1: Intraclass Correlation Coefficients between in-home and in-person assessments for comparison with published literature. | | | | | |
| --- | --- | --- | --- | --- | --- |
|  | Intraclass Correlation Coefficient | F | p value | Lower bound | Upper bound |
| Motor UPDRS | 0.757 | 4.117 | <0.001 | 0.464 | 0.891 |
| Total UPDRS | 0.775 | 4.720 | <0.001 | 0.505 | 0.898 |
| MoCA | 0.825 | 5.539 | <0.001 | 0.607 | 0.922 |
| RBD-Q | 0.870 | 7.464 | <0.001 | 0.709 | 0.942 |
| ESS | 0.860 | 6.926 | <0.001 | 0.686 | 0.937 |
| PDQ-39 | 0.839 | 7.220 | <0.001 | 0.613 | 0.930 |
| TUG | 0.789 | 4.731 | <0.001 | 0.533 | 0.905 |

| Supplementary Table 2 – Post-hoc analysis comparing those preferring in-person visits to those that did not report such preference. | | |
| --- | --- | --- |
|  | Preference for in-person (n=14) | No preference for in-person (n=36) |
| Sex (Female/male) | 9/5 | 21/15 |
| Education (years) | 16.4 ± 1.7 | 16.3 ± 2.4 |
| Race (Caucasian %) | 100% | 100% |
| Age at enrollment (years) | 64.3 ± 8.7 | 66.4 ± 9.5 |
| Disease duration (years) | 7.6 ± 4.1 | 9.8 ± 6.2 |
| Hoehn & Yahr stage | 1.9 ± 0.5 | 2.0 ± 0.5 |
| Modified motor UPDRS | 14.3 ± 7.0 | 11.8 ± 6.2 |
| Modified total UPDRS | 24.4 ± 10.4 | 23.8 ± 10.9 |
| MoCA score | 25.7 ± 2.6 | 26.3 ± 3.0 |
| N-FOG-Q score | 12.5 ± 7.3 (n=4) | 14.7 ± 6.4 (n=13) |
| PDQ-39 | 20.3 ± 14.7 | 30.3 ± 23.0 |
| RBD-Q | 3.1 ± 2.2* | 5.7 ± 3.2 |
| Epworth Sleepiness Scale | 6.9 ± 4.8 | 8.0 ± 5.0 |
| Daily levodopa dose (mg) | 616 ± 251 | 700 ± 341 |
| On agonist/MOA-I | 14%/36% | 33%/42% |
| Mean 10ft TUG time (s) | 12.1 ± 4.0 | 11.8 ± 2.7 |
| Distance from UAMS; 25/50/75 percentile (miles) | 8/36/107 | 10/44/107 |
| Participant survey: | | |
| Scheduling appointment was easy:  Strongly agree  Somewhat agree  Neither agree nor disagree  Somewhat disagree  Strongly disagree | 86%  14%  0%  0%  0% | 86%  14%  0%  0%  0% |
| I was happy with my telemedicine visit: Strongly agree  Somewhat agree  Neither agree nor disagree  Somewhat disagree  Strongly disagree | 71%^#^  21%  0%  7%  0% | 100%  0%  0%  0%  0% |
| What did you like about the telemedicine visit:  No travel arrangements  Ability to be in comfort of your home  Ability to participate in research | 64%  64%  79% | 72%  92%  83% |
| What did you dislike about the telemedicine visit:  Poor video connection  Unable to hear provider  Poor internet connection | 7%  21%  6% | 6%  22%  6% |
| More likely to participate in telemedicine research in the future:  Strongly Agree  Somewhat Agree  Neutral  Somewhat Disagree  Strongly Disagree | 0%^#^  43%  43%  7%  7% | 40%  29%  29%  0%  3% |
| Whom do you rely on for in-person visits? (check all that apply)  Self  Spouse  Children  others | 71%^  43%  0%  0% | 61%  56%  8%  3% |
| Whom did you rely on for telemedicine visit? (check all that apply) Self  Spouse  Children  others | 79%^  29%  0%  14% | 81%  36%  3%  3% |
| Overall visit rating: extremely bad  bad  neutral  good  excellent | 0%^#^  0%  0%  64%  36% | 0%  0%  0%  11%  89% |
| Annual Income: <$25,000  $25-50,000  $50-75,000  $75-100,000  >$100,000 | 0%  0%  27%  9%  64% | 6 %  22%  13%  16%  44% |
| Costs to attend in-person visit <$35  $36-75  $76-150  $151-300  >$300 | 55%  18%  9%  9%  9% | 56%  19%  13%  9%  3% |
| ^#^p<0.05 by Mann-Whitney U-Test  ^p<0.05 repeated-measures analysis for self-reliance between in-person and telemedicine visit. | | |
